# More than Anecdotes: Fishers’ Ecological Knowledge Can Fill Gaps for Ecosystem Modeling

**DOI:** 10.1371/journal.pone.0155655

**Published:** 2016-05-19

**Authors:** Ana Helena V. Bevilacqua, Adriana R. Carvalho, Ronaldo Angelini, Villy Christensen

**Affiliations:** 1 Institute for the Oceans and Fisheries, University of British Columbia, Vancouver, BC, Canada; 2 Ecology Department, Federal University of Rio Grande do Norte, Natal, RN, Brazil; 3 Civil Engineering Department, Federal University of Rio Grande do Norte, Natal, RN, Brazil; University of California Santa Cruz, UNITED STATES

## Abstract

**Background:**

Ecosystem modeling applied to fisheries remains hampered by a lack of local information. Fishers’ knowledge could fill this gap, improving participation in and the management of fisheries.

**Methodology:**

The same fishing area was modeled using two approaches: based on fishers’ knowledge and based on scientific information. For the former, the data was collected by interviews through the Delphi methodology, and for the latter, the data was gathered from the literature. Agreement between the attributes generated by the fishers’ knowledge model and scientific model is discussed and explored, aiming to improve data availability, the ecosystem model, and fisheries management.

**Principal Findings:**

The ecosystem attributes produced from the fishers’ knowledge model were consistent with the ecosystem attributes produced by the scientific model, and elaborated using only the scientific data from literature.

**Conclusions/Significance:**

This study provides evidence that fishers’ knowledge may suitably complement scientific data, and may improve the modeling tools for the research and management of fisheries.

## Introduction

Ecological models have been advocated by the Food and Agriculture Organization of the United Nations (FAO) as promising methodologies for ecosystem-based assessments aimed at managing fishing resources [1,2]. Ecosystem-based fisheries management (EBFM) focuses on a multi-species approach as part of an integrated vision of the ecosystem, rather than on single target populations or fishing fleets [3,4]. Therefore, it should include ecological, social and economic aspects [5], and simultaneously, consider fish, fishers, the maintenance of fishery resources, and the environment [6]. Ecosystem modeling may provide the analytical tool for achieving these goals by considering the ecosystem as a whole, and underpinning the direct and indirect effects of a specific fishery on target and non-target species, and on other fisheries [7].

Ecosystem modeling in fisheries is often hampered by a shortage of consistent and regular fishing statistics [8]. At the same time, the models are also susceptible to the lack of local and quantitative ecological data [9]. Fisheries, on the other hand, are often data limited, markedly so in developing countries, which have no current mandatory fishery statistics [10]. As a result, roughly 99% of fisheries or fish stocks worldwide are unassessed, and remain data-poor fishery scenarios. Beyond that, many of the most data-poor fishing grounds are also the world’s most vulnerable and rapidly changing ecosystems [11].

In contrast, fishers can provide broad and detailed information on fish species and ecology [12], habitats [13], and the environment as a whole [14], including its past and recent changes [15,16,17]. Fishers also retain important information on fishing activity, such as species composition, catches, and abundance [11,18]. This critical data availability with regard to fisheries has led to increasing interest in the place-based knowledge of resource users [19,20], increasing the use of fishers’ ecological knowledge (FEK) [11], or simply FK, as we refer to here.

FK is generated by a fisher’s observation of their fishing environment [21], and by interactions with and observations of other fishers [22]. Its use has been increasingly recognized as a good source of data [12], which has a low cost and is easily sampled by simple methods [11]. However, a fisher’s knowledge must be used with caution, since fishers, like everybody else, may miss or forget information [12]. Moreover, the costs of data collection vary under different circumstances, and data reliability should be tested whenever possible [10]. Aiming to appropriately collect, analyze, and use fishers’ knowledge, researches have recently focused on methodological issues for using FK, defining a suitable sample size, and using the proper variance in the samples, underscoring the awareness of the suitability of the data registered [11].

For modeling and management purposes, the use of FK associated with science could enhance knowledge and user participation, by sourcing the most experienced and, therefore, expert fishers in the fishery and the environment [15,22]. Notwithstanding the evidence on the validity of FK, and its growing employment to fill the gaps in the scientific knowledge [12,13,15,16,18,23], fishers’ knowledge is sometimes referred to as “anecdotal knowledge”, likely limiting its translation into scientific knowledge, the development of methods to properly sample this knowledge, and its application to management [21]. As a result, the inclusion of FK into fisheries management remains the exception, rather than the rule [14]. Following the slow pace for new ideas, FK has thus far not been included in data sampling aiming to model fisheries, despite the scientific recommendations [24].

As a first step to using FK in the ecosystem modeling approach, we interviewed fishers to estimate the parameter values for a model of a coastal fishing area. We used one of the most widely applied modeling approaches and software, Ecopath with Ecosim (EwE) [8], to analyze their contribution to the modeling process, while describing the marine fishing environment, its food web, and the fisheries. The main questions addressed were: Could an Ecopath model be elaborated using fishers’ knowledge? If so, which variables are fishers more capable of informing? Do the attributes generated in the FK model match those of a scientific-based model? Is it possible to improve a food web model sourcing fishers as experts to complement the information? The main expectation here is to compare the ecological role of the relevant species between the two models, investigate the ecosystem attributes, identify the lack of information that could be provided properly by fishers, and determine what should be focused on by the scientific community. We hope to underscore the role of fishers and science as partners to inform fisheries, enhancing managers’ understanding of the fisheries-related food web.

This paper is structured as follows: The FK and science-based (SC) information is discussed within the context of fishing ecosystem modeling. Then, the FK data collection is described, focusing on the methods for selecting interviewees (our experts), and for consulting them. Similarly, the approach used for collecting the science-based data is presented. Next, the methodology used to build the Ecopath model and the output analyses involved are summarized. The attributes of the FK model and SC model are compared, and the paper is concluded with a view on the potential of fishers providing information for modeling and the role of science in filling the knowledge gaps. The aim was to show the value of this new approach to model fishing ecosystems and to better contribute to the understanding, studies, and future management of fisheries.

### Study Area

**Fig 1:**
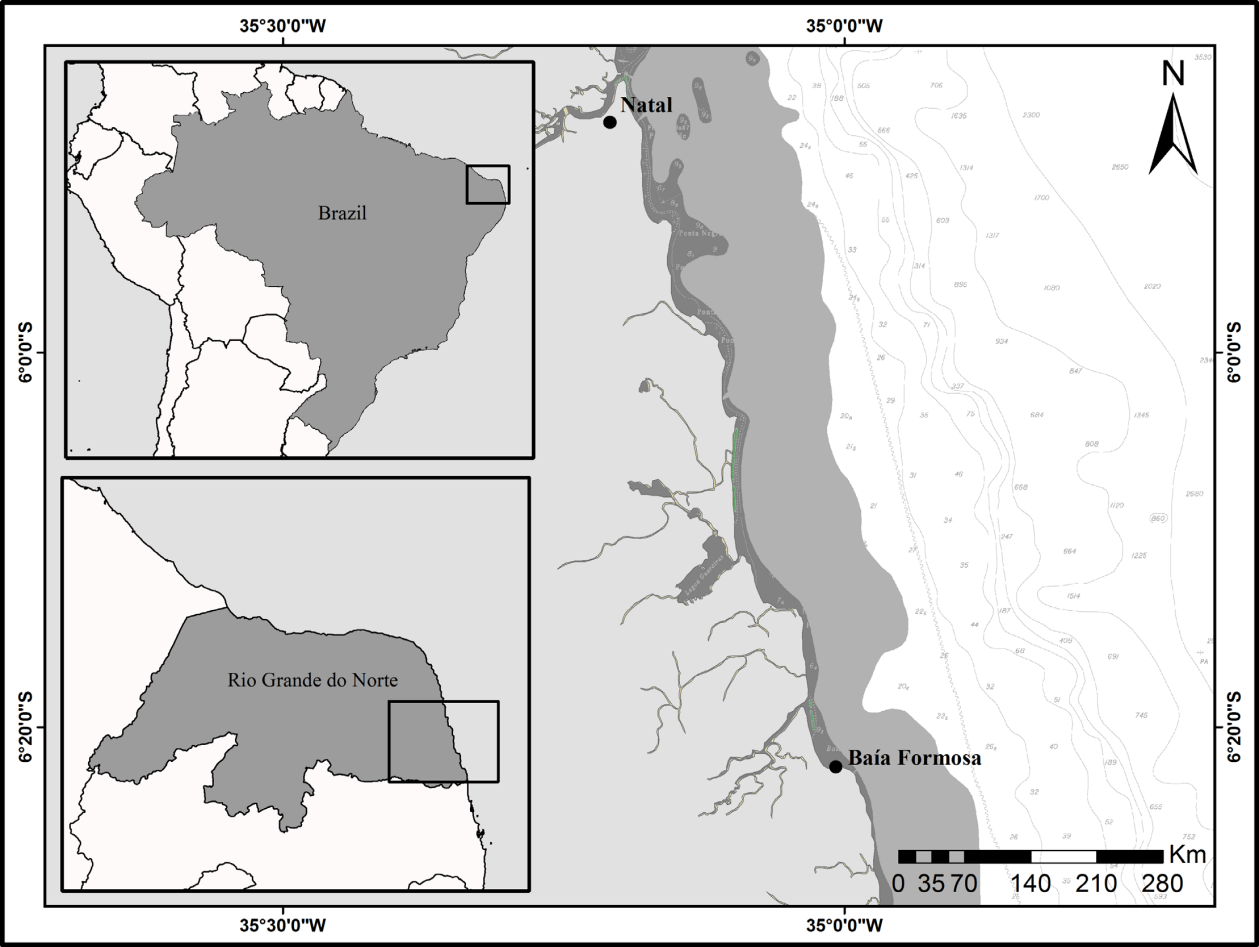
Study area of the fishery community (Baía Formosa) on the Brazilian northeast coast.

The study area included the small-scale fisheries at the continental shelf in the Rio Grande do Norte state, on the Brazilian northeast coast, corresponding to the FAO area “Northern-Natal division 41.1.2” (Fig 1). In this region, the continental shelf break occurs approximately 30–40 km from the coast, usually at a depth of 60–80 m [25]. The small-scale fisheries occur over an area of approximately 16,400 km^2^, with substrates characterized by reef coral sediments and calcareous algae (6 km from coast, and 20 m in depth), showing a high diversity and low abundance of fishes and invertebrates.

Small-scale fisheries in Rio Grande do Norte catch around 11,500 tons per year, which corresponds to 80% of the total catch of this state [26]. The fishery activities are distributed over 95 coastal communities in Rio Grande do Norte, and directly provide employment for 12,300 fishers [25]. Hand lines and gillnets are the most commonly used gear, but the fishers also use traps and diving, as well as trawlers and diving for invertebrates. The fishing fleets operating in this state include sail or paddle powered (about 3,750, or 80% of the total number of boats), motorized (about 900, or 20% of the total), and industrial (about 50, or 1.5%) [27].

The major species in the official landing statistics include tuna and tuna-like species (*Thunnus atlanticus* and *Euthynnus alletteratus*), mackerel (*Scomberomorus brasiliensis* and *S*. *cavalla*), grouper (*Mycteroperca bonaci*), and snapper (*Lutjanus analis* and *L*. *jocu*). Invertebrate catches are also economically important, and include octopus (*Octopus insulares*), lobster (*Panulirus argus* and *P*. *laevicauda*), and shrimp (*Litopenaeus schmitti*, *Xiphopenaeus kroyeri*, and *Penaeus brasiliensis*) [27].

## Material and Methods

**Modeling and data used.** Two ecosystem models were constructed for the same fishing area using the Ecopath with Ecosim software: one model based on the fishers’ knowledge and one model based on the available scientific information. For the first model, the data were collected by interviews, and for the second, the data were gathered from the scientific literature.

In both models, we used the same landing data provided by the Brazilian Environmental Agency (IBAMA). From 2001 to 2011, the artisanal catches for each species or functional group were selected. The catch data included in the model was restricted to the landings, and does not include the estimate of the discards or catches from other fishing activities (recreational, subsistence, research, or illegal). The landing data were expressed in tons (t) per square kilometer per year.

Due to the lack of information on the fishing area of focus, the biomass was predicted based on catches using the Vasconcellos and Cochrane model [28] for both Ecopath with Ecosystem (EwE) models. This model predicts the biomass dynamics according to the Schaeffer model:

$$B_{t}=B_{t-1}+{rB}_{t-1}\left(\frac{1-B_{t-1}}{K}\right)-C_{t-1}$$

where *B* is the biomass, *r* is the intrinsic rate of the increase in biomass and *K* is the virgin biomass or stock at the arraying capacity [28]. The *K* parameters were obtained from fishbase.org, and the catch (*C*_*t-1*_) data are described above. Finally, the analyses were carried out using R software, specifically, the COM-SIR package.

### Fishers’ Knowledge Model (FK Model)

#### Selecting interviewees

All of the interviewed fishers live in the fishing community of Baía Formosa, the most productive fishing community in the austral portion of the state. This community has roughly 200 fishers, and all of them land their catches at Beach Porto, where the landings were sampled.

Only those fishers with higher catches were approached to provide (mainly) the information unavailable in the scientific literature. The interviewee choice was guided by the landing data previously registered from February of 2013 through February of 2014. Based on the landing data registered by the research team during this one-year period, we identified the ten most landed fish species. The recorded landings further enabled us to identity the fishers with higher catches, when using one of the two main types of gear (hand lines or gillnets). For the purpose of this study, these were considered to be the expert fishers that should be consulted.

In addition to having higher fishing productivity, the expert fishers also needed to fulfill pre-established criteria to be considered in this survey: (1) willingness and availability to participate in the research, (2) fishing experience (in years), and (3) being an elder, assuming that older fishers had fished for a longer period of time and would have greater ecological knowledge [29].

#### Method for consulting expert fishers

After the aforementioned selection of experts, 23 fishers were individually selected and interviewed face-to-face in the Baía Formosa community, between February and April of 2014. The same researcher conducted all of the interviews, and the expert fishers were asked for ecological information about the ten most often caught fish species.

The interviews were conducted following an adaptation of the Delphi methodology [30], seeking to add value to the local knowledge by achieving consensus among experts over several rounds of investigation. This method is driven by the assumption that combining the expertise of several individuals will provide more accurate results than what can be achieved from consulting a single individual only [14]. The fishers may have been interviewed under many rounds of consultation, aiming to achieve consensus on the information provided by them. Then, the results of all of the responses in each round were summarized and presented individually to each participant, with the purpose of finding consensus among them. Consensus occurred when at least 50% of the experts reached the same conclusion and agreed on the results. Then, the data were based on individual knowledge and consensus, avoiding bias due to the presence of a group (known as social desirability) or groupthink [30].

**Fig 2:**
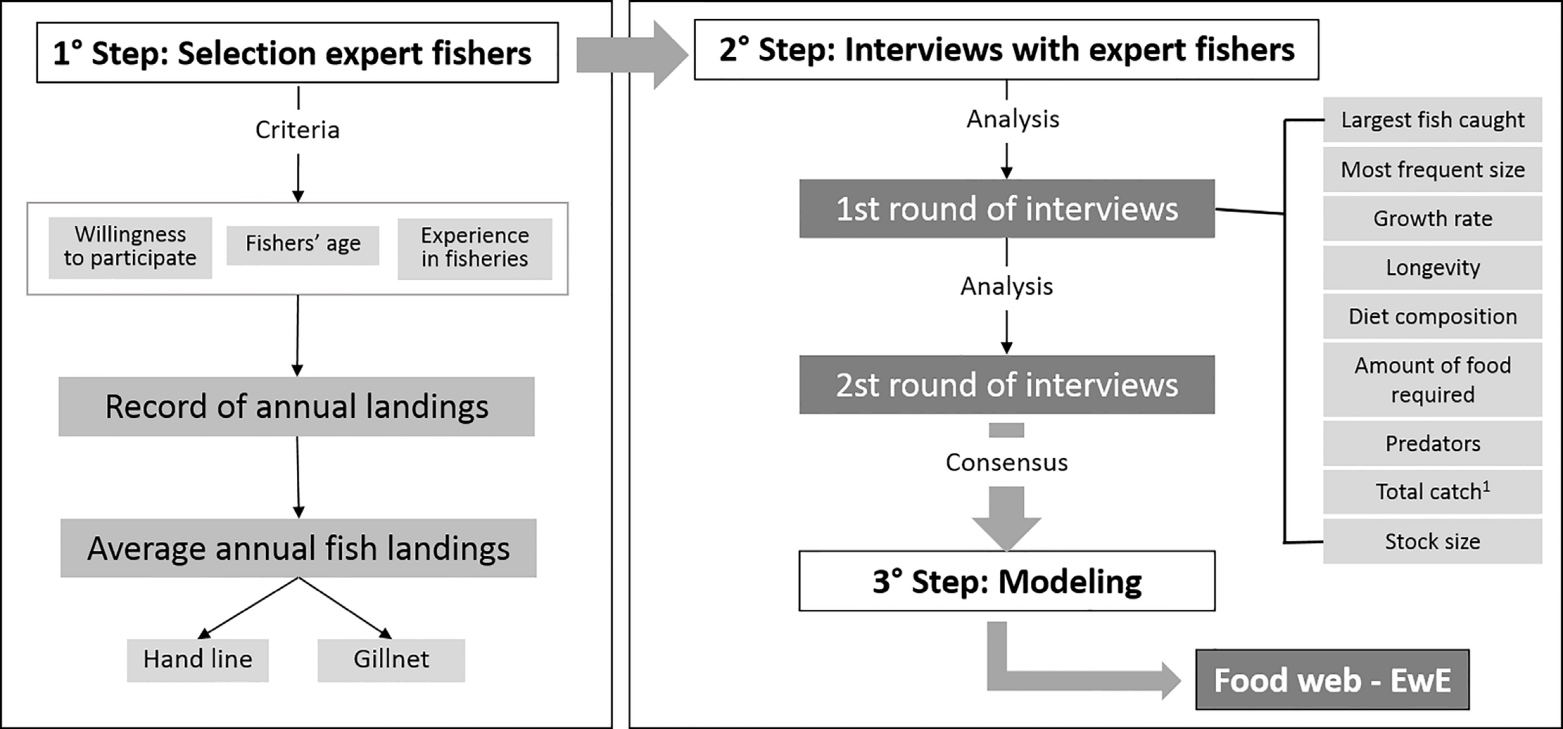
Interviewing methodology: Steps for interviewing methodology applied to register fisher knowledge aiming to select expert fishers and to fill the gaps in the scientific data needed for modeling.

Here, were used two rounds of interviews (Fig 2): in the first one, the individual expert fishers could choose one fish species to address. After that, other fish species were presented (in color photographs) and the fisher could discuss them. At this step, the interviewer showed the information from the first round (from other fishers), and each fisher could review his responses according to the new general data. As stated before, the information was considered consensual when confirmed by more than 50% of the fishers.

Considering the fact that the fishers could not follow the goals of the scientists with regard to the research, the research team conducted two previous meetings with the fishers in the middle of 2013, which had theoretical and practical components. During the first meeting, the fishers were presented with scientific studies on fisheries ranging from unispecific to ecosystem-based evaluations. A few months later, they took part in a participatory approach to learning and evaluating a fishery resource, stock assessment, and management. Moreover, the research team has sampled landing data with the collaboration of local fishers since January of 2013. This scenario has enabled the interviews with the fishers, increasing their involvement with and understanding of the scale and scope of this study.

All of the fishers approached were first informed about the research purpose; then, they were asked about their willingness to participate in this study. Only those who gave verbal consent were included, and their participation was always witnessed by a third person from the university, who was not involved in the research. Despite sounding unusual, oral consent has been accepted and approved by ethics committees in previous research [16]. Oral consent is an instrument that allows the inclusion of illiterate interviewees and/or traditional communities who are not comfortable with tape recording. Actually, there was a high proportion of illiteracy among the interviewed fishers, and to register their consent to participate, the fishes were asked to mark a specific place on the consent form or write their name, if they were formerly trained in this for other purposes.

The data sampling was approved according to the guidelines of the Committee of Ethics at the Federal University of Rio Grande do Norte (Protocol of approval No. 48378415.9.0000.5537). The rules of approval state that the data and metadata related to personal interviews may be available under a specific request and justification, aiming to protect interviewees who may have provided private information.

#### Registering Fishers’ Knowledge and estimating parameters

During the above-mentioned consultation of fishers, the information registered included population parameters like the (1) maximum weight (W_max_) and/or length (L_max_), (2) modal weight (W_modal_) or length (L_modal_), (3) growth rate, and (4) longevity, as well as trophic information like the (5) diet composition, (6) amount of food required per day, (7) main predators, (8) total catch per species during the last year, and (9) total stock size. The details about the data collection instruments are available in the Supporting Information (S1 Table).

The expert fishers were presented with pictures of food items in order to assist them when providing information about their diet items. Even when using the pictures, the interviewees added other species as diet items, providing detailed information about their diet compositions (S3 Table). In addition, the expert fishers could use pieces of graduated tape to show their perceptions of the estimation of length (L_max_ and L_modal_ in cm). In the cases in which the fishers provided the fish dimensions in weight (kilograms), the length (L∞) was estimated by weight-length relationships using an online database (fishbase.org). The fishers provided information on the L_max_, which was used to estimate the asymptotic length (L_∞_) for each species, based on the empirical equation of Froese and Binohlan [31].

The fishers’ knowledge data provided information to estimate the Natural Mortality through Pauly’s equation [31], which was assumed to be equal to the production/biomass (P/B). The weight estimates based on the fishers’ knowledge were used to calculate the consumption rate (Q/B), using the Palomares and Pauly equation [32].

During the interviews, in addition to being asked about the stock sizes for the ten most caught fish species, the fishers were provided with corn seeds that they could use to represent the quantity of each species they caught annually. They were encouraged to add as many seeds as they needed to coarsely represent the total size of each fish stock. However, the fishers were unable to provide accurate information on the annual catch or the stock sizes, by any means.

### Science data-based Model (SC Model)

Aiming to provide information for all of the compartments in the scientific model, data was gathered from scientific papers, reports, theses, governmental reports, online databases, and other EwE models created in Brazil or in other tropical ecosystems [33–36].

As in the FK model, the P/B for the fish species was calculated based on Pauly’s equation [37], and the Q/B was estimated using the method of Palomares and Pauly [32]. For the non-fish compartments, we used Gascuel et al. [38] for the P/B estimates, and multiplied them by three for the Q/B. All of the basic input parameters were expressed per unit of surface area, that is, in tons per square kilometer. The basic input estimates for each species were used to calculate an average value of that basic input for each functional group.

The diet matrix was summarized as the percentage of each group in terms of the total wet weight (or volume) in the diet of the predator. The diet composition matrix was determined using an online database (fishbase.org) and other studies of the same group or species and region. The main studies consisted of dolphins [39–41], sharks [42–45], dogfish [46], large pelagic fish [43,47], *Thunnus atlanticus* [48], grouper [49], *Lutjanus* spp. [50], *Coryphaena hippurus* [43], *Euthynnus alletteratus* [51], octopus and squid [52–54], carnivorous zoobenthos [55–57], detritivorous zoobenthos [57,58], and *Scomberomorus brasiliensis*, *Seriola fasciata*, *Cynoscion jamaicensis*, and *S*. *cavalla* (fishbase.org) (S3 Table).

### Model Description

EwE version 6.4.3 was used to elaborate both models (FK and SC). This modeling tool was initially developed by Polovina [59], and has been further developed by Christensen and Walters [60]. EwE enables the integration of a large body of information into a coherent description of an aquatic food web, while accounting for human activities in an ecosystem context, and environmental changes [8].

The Ecopath model is built on a system of linear equations to describe the average flows of mass and energy between a series of functional groups that represent the organisms in a food web. The flow to and from each functional group is described by the main Ecopath equation representing the production of each group *i*:

$$B_{i\;}\left(\frac{P}{B_{i}}\right){EE}_{i}=\sum B_{j}\;\left(\frac{Q}{B_{j}}\right)\;DC_{ji}\;+\;Y_{i}\;+\;E_{i}+{BA}_{i}$$

where *B*_*i*_ is the biomass of *i*, *P/B*_*i*_ is the production/biomass ratio, *EE*_*i*_ is the ecotrophic efficiency, *B*_*j*_ is the biomass of the consumers or predators *j*, *Q/B*_*j*_ is the consumption per unit of biomass of *j*, *DC*_*ji*_ is the fraction of the prey *i* in the diet of predator *j*, *Y*_*i*_ is the total fishery catch rate, *E*_*i*_ is the net migration rate, and *BA*_*i*_ is the biomass accumulation rate [60].

In order to parameterize the model, three out of four basic parameters must be provided for each functional group: biomass (B), production per unit of biomass (P/B), consumption per unit of biomass (Q/B), and ecotrophic efficiency (EE). The algorithm then estimates the fourth parameter to ensure the mass balance. For each group, only one of the basic input data could be missing, which was then estimated by the model [61]. The diet composition and landings are also required.

#### Output Analysis

For this analysis, we estimated the ranking correlation between the 10 main species in the landing data (in tons) and other catch species according to the fishers’ interviews, using the non-parametric Spearman index. The paired t test was used to compare the input parameters, P/B, and Q/B between the models, assuming that each interview could be treated as an independent sample.

A paired t test was also used to compare the trophic level (TL), omnivory index (OI), and keystone index (KS) to each of the main fish species in both EwE models. The TL provides an estimate of the trophic position of a particular species or functional group in the food web. The OI measures the distribution of the feeding interactions among the trophic levels by functional group [61], while the KI estimates the effects of each group on all of the other groups in the food web, including the indirect ones [62].

The attributes used to compare the outputs in both models (FK and SC) were the total production, total throughput system (total flow, including detritus), and total net primary production. The general attributes of the ratios used to evaluate the matches between the models were the total primary production/total respiration, total primary production/total biomass, and total biomass/total throughput. The total biomass (excluding detritus) and total catch were used to compare the biomasses. We also compared the mean trophic levels of the catch. The main indexes to measure the resilience and stability in each model were the connectance, system omnivory, ascendancy, overhead, Finn cycling index, and Finn mean path length [60].

## Results

Between February and April of 2014, 23 fishers were interviewed (12 hand line users and 11 gillnet users). Their ages ranged from 26 to 57 years old (median = 41 years old and mode statistic = 35 years old), and their mean age when starting in the fishery was 14 years old. Their fishing experiences varied from 10 to 45 years, with an average of 24 years. Altogether, they represented 470 years of fishing experience.

The most commonly caught species according to the interviews were *Lutjanus analis* (mutton snapper, 17.1%), *Scomberomorus brasiliensis* (Spanish mackerel, 14.6%), and *Thunnus atlanticus* (blackfin tuna, 12.2%). *Euthynnus alletteratus* (Atlantic little tunny) and *Rhizoprionodon lalandii* (dogfish) were mentioned by 9.8% of the fishers. Each one of the following species *Coryphaena hippurus* (dolphinfish), *Cynoscion jamaicensis* (weakfish), *Mycteroperca bonaci* (black grouper), *Scomberomorus cavalla* (king mackerel), and *Seriola fasciata* (lesser amberjack) were referred to by 7.3% of the fishers. Moreover, the fishers reported all of the aforementioned species as the ten most caught fish species.

The correlation between the catches and frequency in which the species were chosen by each fisher during the interviews (Spearman index ρ = 0.83) shows how confident the fishers were when talking about the species with which they were most familiar and had greater knowledge, that is, species with higher landings.

### Basic and Output Parameters

**Fig 3:**
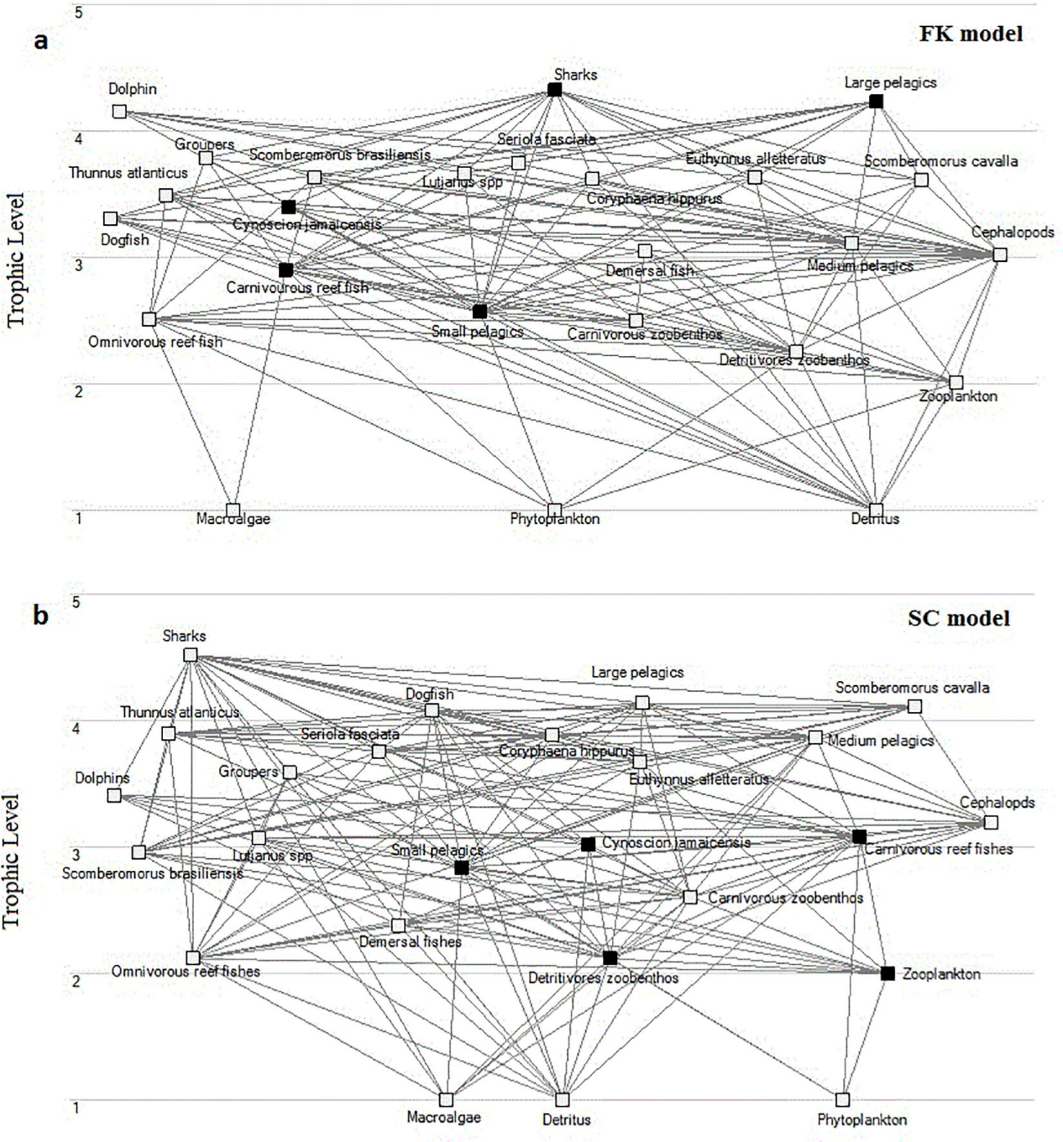
Modeling of trophic network by Ecopath with Ecosim for the fishing habitat of Baía Formosa (Brazil). (A) Fisher knowledge model and (B) science-data model as input parameters. Black square: keystone groups.

A total of 25 compartments represented the ecosystem in both models (FK and SC). Each compartment was expressed by either a single species or functional groups, and was comprised of ecologically related species, depending on their importance in the landing records (Table 1, Fig 3).

**Table 1:** Basic input for the EwE to the FK model and SC model. B = biomass (wet weight–t*km^-2^); P/B = production/biomass (year^-1^); Q/B = consumption/biomass (year ^-1^); EE = ecotrophic efficiency. Species aggregation in each compartment. FK model = fishers’ knowledge model; SC model = science-data model.

	Biomass	P/B to each model		Q/B to each model		EE to each model		Species aggregation
Group		SC	FK ^5^	SC	FK ^9^	SC	FK	
Dogfish	0.055 ^2^	0.84 ^6^	0.61	5.70 ^10^	6.46	0.43	0.37	*Rhizoprionodon lalandii*, *R*. *porosus*
*Thunnus atlanticus*	1.200 ^3^	0.54 ^6^	0.53	7.22 ^9^	9.50	0.45	0.66	*Thunnus atlanticus*
Grouper	0.478 ^3^	0.74 ^6^	0.35	3.20 ^10^	3.92	0.59	0.16	*Epinephelus adscensionis*, *E*. *itajara*, *Mycteroperca bonaci*
*Scomberomorus brasiliensis*	1.243 ^3^	1.11 ^6^	0.32	5.70 ^10^	9.22	0.60	0.82	*Scomberomorus brasiliensis*
*Lutjanus* spp	2.291 ^3^	0.87 ^6^	0.44	4.90 ^10^	6.32	0.11	0.65	*Lutjanus analis*, *L*. *jocu*, *L*. *synagris*
*Seriola fasciata*	0.430 ^4^	0.52 ^5^	0.42	3.81 ^10^	4.51	0.98	0.91	*Seriola fasciata*
*Coryphaena hippurus*	0.672 ^3^	1.78 ^5^	1.66	3.80 ^9^	5.07	0.46	0.66	*Coryphaena hippurus*
*Euthynnus alletteratus*	0.397 ^3^	0.77 ^6^	0.43	7.10 ^10^	11.25	0.92	1.00	*Euthynnus alletteratus*
*Cynoscion jamaicensis*	0.195 ^3^	0.56 ^6^	0.64	7.30 ^10^	4.48	0.68	0.18	*Cynoscion jamaicensis*
*Scomberomorus cavalla*	0.250 ^3^	0.81 ^6^	0.29	4.50 ^10^	12.67	0.67	0.92	*Scomberomorus cavalla*
Dolphins	0.014 ^1^^-^	0.68 ^5^		35.50 ^2^		0.13	0.00	*Pontoporia blainvillei*, *Sotalia guianensis*, *Tursiops trucatus*
Sharks	0.064 ^2^	0.26 ^5^		3.57 ^10^		0.32	0.00	*Carcharhinus brevipinna*, *C*. *limbatus*, *C*. *longimanus*, *C*. *obscurus*, *C*. *signatus*, *Galeocerdo cuvier*, *Isurus oxyrinchus*, *Prionace glauca*, *Sphyrna lewini*, *S*. *zigaena*
Large pelagics	0.510 ^2^	0.39 ^5^		7.30 ^10^		0.58	0.06	*Istiophorus albicans*, *Katsuwonus pelamis*, *Makaira nigricans*, *Tetrapturus albidus*, *Thunnus alalunga*, *T*. *albacares*, *T*. *obesus*, *Xiphias gladius*
Medium pelagics	1.600 ^1^	0.63 ^5^		5.06 ^10^		0.98	0.96	*Centropomus parallelus*, *C*. *undecimalis*, *Elagatis bipinnulata*, *Rachycentron canadum*, *Sphyraena barracuda*
Small pelagics	4.475 ^2^	4.41 ^1^		12.71 ^9^		0.72	0.96	*Sardinella brasiliensis*, *S*. *crumenophthalmus*, *Selar crumenophthalmus*, *Trachurus lathami*
Carnivorous reef fishes	4.630 ^8^	0.63 ^5^		6.37 ^10^		0.78	0.93	*Archosargus rhomboidalis*, *Caranx bartholomaei*, *C*. *latus*, *Dermatolepis inermis*, *Elops saurus*, *Gymnothorax vicinus*, *Haemulon plumieri*, *H*. *steindachneri*, *Ocyurus chrysurus*, *Oligoplites saurus*, *Pseudupeneus maculatus*
Omnivorous reef fishes	4.670 ^8^	1.44 ^5^		8.52 ^9^		0.88	0.69	*Balistes vetula*, *Holocentrus adscensionis*, *Myripristis jacobus*, *Scarus trispinosus*, *Sparisoma rubripinne*, *S*. *viride*
Demersal fishes	2.500 ^2^	0.68 ^5^		7.50 ^10^		0.75	0.15	*Bagre bagre*, *Dasyatis guttata*, *Dasyatis spp*, *Micropogonias undulatus*, *Orthopristis ruber*, *Pomadasys corvinaeformis*, *P*. *formis*, *Selene volmer*, *Umbrina canosai*
Cephalopods	3.800 ^2^	1.88 ^9^		10.7 ^2^		0.83	0.98	*Octopus insularis*, *Loligo* spp.
Carnivorous zoobenthos	5.700 ^1^	2.50 ^5^		10.00 ^1^		0.96	0.91	*Chaceon* spp., *Panulirus argus*, *P*. *Iaevicauda*, Equinoderm
Detritivorous zoobenthos	13.430 ^4^	2.61 ^5^		13.02 ^1^		0.75	0.75	*Farfantepenaeus brasiliensis*, *Litopenaeus schmitti*, *Xiphopenaeus kroyeri*, Crustacea, Equinoderm, Mollusca, Polichaeta
Zooplankton	14.078 ^2^	40.00 ^7^		165.00 ^1^		0.16	0.25	Chlorophyta, Cryptophyceae, Diatomophyceae, Euglenophyceae, Xantophyceae
Macroalgae	98.450 ^1^	13.25		-		0.05	0.02	Chlorophyta, Rhodophyta, Phaeophyta
Phytoplankton	35.000 ^4^	70.00 ^1^		-		0.97	0.97	Amphipoda, Appendicularia, Chaetognatha, Cladocera, Coelenterata, Copepoda, Ctenophora, Euphausiaceae, Mysidaceae, Pteropoda, Rotifera, Thaliacea, Turbellaria
Detritus	201.910 ^1^	-		-		0.06	0.29	-

^1^ [33]

^2^ [34]

^3^ [28]

^4^ EwE estimation

^5^ [37]

^6^ [38]

^7^ [35]

^8^ [36]

^9^ [32]

^10^ fishbase.org.

The expert fishers were unable to answer questions about longevity, total catch, and stock size, or about the amount of food required per day, which they overestimated by more than 100 times that expected by the fish metabolism (Table 2). For example, according to the fishers, the piscivorous species would eat 3 times their own weight in a day. Therefore, this, last information was not used for the modeling, and the W_max_ allowed us to calculate the W_∞_ used to estimate the Q/B for the FK model [32].

**Table 2:** Average values of fishers’ knowledge for the maximum (W_max_) and modal (W_modal_) sizes, and amount of food required per biomass per year. The weight-length relationships were used to estimate the length (L∞) from the related maximum weight. The values in the brackets refer to the standard deviation.

Group	W_max_ (g)	W_modal_ (g)	L∞ (cm)	Food required (gr/year)
Dogfish	4,333	2,500	104.48	165.77
	(1,154)	(707)	(10.53)	(190.71)
*Thunnus atlanticus*	12,300	5,400	104.84	69.77
	(2,729)	(547)	(8.23)	(72.16)
Grouper	43,000	20,000	149.71	38.23
	(7,000)	(14,142)	(8.01)	(15.9)
*Scomberomorus brasiliensis*	5,250	2,333	95.38	19.46
	(758)	(605)	(4.84)	(24.87)
*Lutjanus* spp.	8,333	3,083	91.04	87.6
	(1,366)	(801)	(4.79)	(28.4)
*Seriola fasciata*	51,333	32,500	159.89	19.65
	(13,012)	(3,535)	(14.93)	(11.91)
*Coryphaena hippurus*	23,333	10,000	151.63	47.65
	(10,408)	(2,000)	(23.80)	(12.29)
*Euthynnus alletteratus*	20,500	1,833	107.52	165.81
	(29,045)	(288)	(56.17)	(128.55)
*Cynoscion jamaicensis*	9,133	4,000	88.59	86.33
	(3,775)	(1,414)	(12.89)	(90.23)
*Scomberomorus cavalla*	36,666	9,000	178.49	36.53
	(7,637)	(2,645)	(12.43)	(32.52)

The growth rates were only obtained from a few of the fishers, and only for some of the pelagic surface species, such as *Thunnus atlanticus*, *Scomberomorus brasiliensis*, *Euthynnus alletteratus*, and *Scomberomorus cavalla*. The information for the total catch in the previous year and stock size were obtained only qualitatively (i.e. higher, the same, or lower). Nevertheless, the expert fishers provided information on the maximum (W_max_) and modal weights (W_modal_) (Table 2).

The FK and SC models did not differ in the P/B (t = 1.28, df = 23, p-value = 0.211) and Q/B (t = -1.92, df = 21, p-value = 0.068) ratios. Once the other data used to estimate the P/B and Q/B were the same for both models, they indicated the likeness between the values of the weights observed by the fishers and sampled scientifically, with regard to the ten most caught species.

For the fishers, the most mentioned predators were sharks (36 times), medium pelagics (23), great barracuda (20), dolphins (13), *S*. *cavalla* (11), and groupers (9). This information was used to build the diet matrix in the fishers’ model. However, the overall diet composition was more detailed in the matrix based on the scientific data (S2 Table). Nonetheless, such itemized information did not generate differences in either the trophic level (t test = -0.10242, df = 20, p-value = 0.9194) or omnivory index (t test = -1.759, df = 20, p-value = 0.1555) between the FK and SC models (Table 3). The keystone species indicated the first five species in the scientific model: carnivorous reef fishes, small pelagics, *Cynoscion jamaicensis*, detritivorous zoobenthos, and zooplankton. In the FK model, the most important species were carnivorous reef fish, small pelagics, large pelagics, and sharks (Table 3).

**Table 3:** Trophic level, omnivory index, and keystone index position obtained by the FK and SC models. The values did not differ between the models for the trophic levels (p = 0.92) and omnivory index (p = 0.15).

Group	Trophic level		Omnivory index		Keystone position	
	FK	SC	FK	SC	FK	SC
Dogfish	3.31	4.08	0.69	0.60	22°	24°
*Thunnus atlanticus*	3.49	3.90	0.85	0.70	19°	12°
Grouper	3.79	3.59	1.14	0.74	20°	18°
*Scomberomorus brasiliensis*	3.63	2.96	0.71	0.07	16°	22°
*Lutjanus* spp.	3.66	3.07	0.93	0.44	17°	6°
*Seriola fasciata*	3.74	3.76	1.08	0.55	18°	21°
*Coryphaena hippurus*	3.62	3.88	0.04	0.58	15°	20°
*Euthynnus alletteratus*	3.63	3.68	0.04	0.71	21°	19°
*Cynoscion jamaicensis*	3.40	3.02	0.38	0.38	4°	2°
*Scomberomorus cavalla*	3.61	4.11	0.12	0.26	23°	11°

In general, the ecosystem attributes (Table 4) were very similar in each model. However, the system omnivory index, throughput cycled (excluding detritus), Finn’s cycling index, and overhead had higher values in the FK model. These differences are related to the different diet compositions used in each model.

**Table 4:** The parameters of the FK model and SC model to the fishing system modeled using EwE.

Parameter	FK model	SC model	SC/FK	Units
Sum of all production	4422.34	4459.84	1.00	t/km^2^/year
Total system throughput	9791.92	8943.97	0.91	t/km^2^/year
Total net primary production	3753.80	3754.46	1.00	t/km^2^/year
Total primary production/Total respiration	2.33	2.46	1.05	-
Total primary production/Total biomass	18.95	19.16	1.01	-
Total biomass/Total throughput	0.02	0.02	1.10	/year
Total biomass (excluding detritus)	198.01	195.91	0.99	t/km^2^
Total catch	0.52	0.48	0.91	t/km^2^/year
Mean trophic level of the catch	2.88	2.76	0.95	-
Connectance index	0.22	0.32	1.40	-
System omnivory index	0.43	0.29	**0.68**	-
Ascendancy (% of capacity)	35.1	54.0	**1.53**	-
Overhead (% of capacity)	64.9	46.0	**0.71**	-
Throughput cycled (excluding detritus)	21.02	0.85	**0.04**	t/km^2^/year
Finn's cycling index	5.87	0.56	**0.09**	% of total throughput
Finn's mean path length	2.547	2.370	0.93	none

## Discussion

Globally, the information provided by the fishers has been used broadly for wide assessments, such the evaluation of fishery resources from the users’ point of view [63], as well as to track changes in fish diversity [15], complement available information on fishing and ecological changes [23], define management units [10], assess ecosystem services through spatial modeling [8], and depict trophic relationships among fishes [52].

With regard to modeling, few studies use fishers’ knowledge, mostly because this knowledge is considered to be anecdotal, non-methodological, and of limited application among scientists and managers [12]. As a result, studies using FK are mostly conceptual and qualitative, overlooking the potential of such data to enhance outcomes from ecosystem modeling under distinct views: from the scientists and from the fishers’ observations. One exception, thus far, is the quantitative modeling approach, which indicates the ability of fishers to report biodiversity losses and food web changes from a historical perspective [15].

Our study is the first to use fishers’ knowledge to parameterize an ecosystem model based on the Ecopath with Ecosim approach. Here, the 19 ecosystem attributes provided by the model which were elaborated using FK were equivalent to the ecosystem attributes produced by the model elaborated using only the data from the literature. Clearly, an EwE model may (partly) be successfully elaborated using the basic parameters provided by fishers. However, the fishers were unable to, even roughly, inform on the growth rates, longevity, biomass, and stock sizes for all of the species. These are precisely the main gaps to modelers in the literature, mainly in developing countries, given the lack of funding and policies to provide these data [8]. Additionally, the information on the food required per day was entirely inconsistent between the two sources of data (the fishers and the literature). When the fishers did not observe the fish during their entire life cycles, they did not understand the rate of growth of those fish. In addition, since they did not see their target fish species feeding, and did not have information about their predation pressure on the prey stocks, they were unlikely to inform on their requirements of food per day.

Conversely, despite the greater detail in the scientific data, the expert fishers were more precise in their information about the maximum and average weights (W_max_ and W_modal_). These parameters tend to be more easily answered by the fishers, since they represent exceptional experiences [18] in our case, in terms of fish size (W_max_), and the expected typical size of each species (W_modal_). Specifically, the W_max_ reminds them on uncommon and pleasant moments, which turn into memories, that are easily recalled.

The fishers were also precise in their information on diet composition, mainly for the ten most caught fish species. Indeed, the FK for all of the species has proven to be a good source of information about important commercial fishes’ diet compositions [13,64,65]. In some cases, however, this information may be based on which prey species they used as bait, and the researcher must be aware of this, and not overemphasize the bait items as food resources [11]. Most importantly, the fishers acquire their knowledge of fish diets gradually, through daily observations of species biodiversity and ecology, or the stomach contents while cleaning the fish that were caught [19].

In this regard, during the interviews, the fishers upheld slightly higher predation rates on small and medium pelagic species, than those reported in the literature. Once it is established that the group of small pelagics is comprised of local species used for the fishers’ consumption, and the group of medium pelagics contains species of interest to recreational fishing, this information is easier to grasp. Beyond that, the small pelagics emerge as keystone species in both the FK and SC models, possibly supporting the fishers’ observations.

With regard to the keystone groups, is interesting to note that the models elaborated upon the observations of the fishers, and the science underscored the compartments of the intermediate trophic level, which includes small pelagics, *C*. *jamaicensis*, and carnivorous reef fishes. What can be said here is, in general, fishers do not bias their information towards fishes of higher economic value, but by simple observation, they guided their modeling to the same outcome that the science would come up with. On the other hand, according to the FK model, the keystone groups would also include top-predators, while the science-based model indicated microinvertebrates. At the current poor data level, these opposed outcomes are conceivable, but challenging, if not impossible, to confirm.

The few discrepancies found here are noteworthy, and may have important management implications. They may indicate minutiae not observed by the fishers (such as the food required per day), and therefore, assumed to be unreal or unimportant. On the other hand, as immediate observers of the sea [23], the fishers may consider the science to be inaccurate if they are confident of specific local evidence (like the higher predation on small and medium pelagics), which is not given by the scientific data. In both of the aforementioned situations, the mismatch between the knowledge revealed by the fishers and the information attained by the science may prompt the fishers to exhibit non-compliance with further management decisions [16].

Currently, there is also a mismatch and low connection between the modeling efforts and their real-world applications, mostly because fishery scientists are the main ecosystem modelers, while the application relies on managers. However, the disconnection EwE modeling has indicated the most threatening fishing gear, the most vulnerable fishing resources or compartments, and the effects of fishing pressure and changes in the environment on the biomass of exploited species [23,66]. All of this evidence guides management suggestions, and the managers may or may not implement them. Regardless of the application of the modeling outcomes, modeling using actual temporal data allows for the proposal of management actions, and its validation under, for instance, changes in the ecosystem [67] or in the fishing pressure.

The proof of consistency among the estimates achieved by both models (FK and SC) is not a new finding, and the agreement between the local fishers’ knowledge and scientific data has been shown previously [12,16,18,23]. The novelty here relies on using fishers’ observations for quantitative modeling, and on revealing that the modeling outcomes were not enhanced or worsened by the fishers’ information. Under this evidence emerges the question of whether only the fishers’ knowledge could be used to model ecosystems in areas that have been poorly studied.

Prompted by the results achieved here, we contend that under dataless scenarios, the use of fishers’ knowledge should not be disregarded. However, simple methodological caution, like carefully selecting the informants when seeking specialized advice, may increase the reliability of the information provided by the fishers [11]. In this case, avoiding random interviews and seeking older fishers is likely to minimize the level of uncertainty [16,23]. This methodology is a practical tool to access FK and conduct ecosystem modeling, and it can be used as a basis for EBFM. One fundamental aspect of EBFM is that it is an interdisciplinary approach, where the fishers’ participation is essential for success [4]. In this sense, including the fishers’ views and experiences may enhance commitment from fishers when establishing management strategies [22].

The use of fishers’ knowledge as a tool to model fisheries is not common, and it is still in its early steps. Fisher experience-based knowledge has been combined with literature and data sampling to understand the loss of biodiversity and food web changes [15], and fishers’ knowledge about the trophic relationships of fishes has been compared with scientific data for providing new hypotheses [13]. In addition, the total catch modeling using the catch per unit of effort estimated by the fishers and by official data has been applied to data evaluation and support management [63].

Despite the consistency of information described by our results, and shown in many articles, fishers and scientists may fail. Therefore, divergences should be treated with similar skepticism, and tested wherever practical, and when scientific and local knowledge diverge, both should be re-examined [11]. The main message remaining here is that some variables may be suitably informed by either the fishers or scientists. Maybe the time has come for science to focus on information that is usually completely unavailable for fish species, such as the life expectancy, growth rate, stock size, and total catch per year, mainly in developing countries. Thus, the main lack of learning relies on the lack of biological and statistics surveys. Improved statistics may, in many ways, enhance fisheries science [68], and here, combined with FK, it could fill the gaps in the knowledge of fishers and scientists, leading to better management of fisheries resources. Furthermore, combining the data from different sources may expand the results, increase the reliability, and be used independently for quick and low-cost assessments.

## Supporting Information

S1 Table Fishers’ questionnaire. Questionnaire that was applied for experts fishers.
(DOCX)

S2 Table Scientific model diet matrix. Diet matrix for the 25 compartments using Ecopath with Ecosim. The predators are set in columns and the prey is set in lines.
(DOCX)

S3 Table Fishers’ knowledge model diet matrix. Diet matrix for the 25 compartments using Ecopath with Ecosim. The predators are set in columns and the prey is set in lines.
(DOCX)
