# Supplementary material for: More than Anecdotes: Fishers’ Ecological Knowledge Can Fill Gaps for Ecosystem Modeling
Source: PLoS One. 2016 May 19;11(5):e0155655. doi: 10.1371/journal.pone.0155655 (PMC4873290; doi:10.1371/journal.pone.0155655)
Supplement: S1 Table — Questionnaire that was applied for experts fishers. (DOCX) [file pone.0155655.s001.docx]

**Supporting information**

**S1 Table. Fishers` questionnaire.** Questionnaire that was applied for experts fishers.

| Just tick as consent for participating | |  | |
| --- | --- | --- | --- |
| Signature as agreement for participating | |  | |
| Interviewer |  | Date |  |
| Fisher/nickname |  | Local |  |
| Age |  | Experience |  |
| **Round 1** | | | |
| 1 – Which fish species were the most caught by you last year? | | | |
| 2 – What was the largest fish of this species you already caught or saw being landed here in this Port? (Use the answer of previous question) | | | |
| 3 – At which size this species is more commonly caught? | | | |
| 4 – How long takes to this fish reach the aforesaid size? (Use previous answer as reference of size) | | | |
| 5 – For how long do you think this fish species can live? | | | |
| 6 – What does this fish species eat? | | | |
| 7 – How much food you believe one fish of this species, at _____of size eats per day? (Here use common size caught, indicated in question 3) | | | |
| 8 – Beyond man, who else eats this fish species? | | | |
| 9 – How much fish do you think this community caught last year? | | | |
| 10 –The landing data we registered here last year in this fishing community indicated that the entire community caught the total amount of _______ (ton or kg) of this species. What is you estimate on the total amount of this fish species caught last year? | | | |
| 11 – How much of this fish species you risk to say is still available in the ocean? | | | |
| **Round 2** | | | |
| 1 – These are the results of interviews for different species that you analyzed in Round 1. | | | |
| 2 - Do you agree with them? Size, catch, food item, etc…(see Round 1) | | | |
