# Supplementary material for: More than Anecdotes: Fishers’ Ecological Knowledge Can Fill Gaps for Ecosystem Modeling
Source: PLoS One. 2016 May 19;11(5):e0155655. doi: 10.1371/journal.pone.0155655 (PMC4873290; doi:10.1371/journal.pone.0155655)
Supplement: S2 Table — Diet matrix for the 25 compartments using Ecopath with Ecosim. The predators are set in columns and the prey is set in lines. (DOCX) [file pone.0155655.s002.docx]

**Supporting information**

**S2 Table. Scientific model diet matrix.** Diet matrix for the 25 compartments using Ecopath with Ecosim. The predators are set in columns and the prey is set in lines.

|  | **Prey \ predator** | **1** | **2** | **3** | **4** | **5** | **6** | **7** | **8** | **9** | **10** | **11** | **12** | **13** | **14** | **15** | **16** | **17** | **18** | **19** | **20** | **21** | **22** |
| --- | --- | --- | --- | --- | --- | --- | --- | --- | --- | --- | --- | --- | --- | --- | --- | --- | --- | --- | --- | --- | --- | --- | --- |
| **1** | Dolphins |  | 0.553 |  |  |  |  |  |  |  |  |  |  |  |  |  |  |  |  |  |  |  |  |
| **2** | Sharks |  | 0.236 |  |  |  |  |  |  |  |  |  |  |  |  |  |  |  |  |  |  |  |  |
| **3** | Dogfish |  | 0.495 | 0.174 |  |  |  |  |  |  |  |  |  |  |  |  |  |  |  |  |  |  |  |
| **4** | Large pelagics |  | 0.175 |  | 0.168 |  |  |  |  |  |  |  |  |  |  |  |  |  |  |  |  |  |  |
| **5** | *Thunnus atlanticus* |  | 0.262 | 0.174 | 0.949 | 0.235 |  |  |  | 0.265 | 0.296 | 0.188 |  | 0.172 | 0.243 |  |  |  |  |  |  |  |  |
| **6** | Groupers |  | 0.262 |  |  |  | 0.947 |  | 0.162 |  |  |  |  |  |  |  |  |  |  |  |  |  |  |
| **7** | *Scomberomorus brasiliensis* |  | 0.270 | 0.174 | 0.149 | 0.420 |  |  |  | 0.265 | 0.296 | 0.188 |  | 0.172 | 0.243 |  |  |  |  |  |  |  |  |
| **8** | *Lutjanus* spp |  | 0.262 |  |  |  | 0.375 |  | 0.126 |  |  |  |  |  |  |  |  |  |  |  |  |  |  |
| **9** | *Seriola fasciata* |  | 0.698 | 0.173 | 0.294 | 0.200 |  |  |  | 0.265 | 0.484 | 0.623 |  | 0.787 | 0.196 |  |  |  |  |  |  |  |  |
| **10** | *Coryphaena hippurus* |  | 0.444 | 0.174 | 0.959 | 0.183 |  |  |  | 0.159 | 0.153 | 0.188 |  | 0.172 | 0.243 |  |  |  |  |  |  |  |  |
| **11** | *Euthynnus alletteratus* |  | 0.262 | 0.174 | 0.171 | 0.584 |  |  |  | 0.150 | 0.195 | 0.743 |  | 0.172 | 0.486 |  |  |  |  |  |  |  |  |
| **12** | *Cynoscion jamaicensis* | *0.497* | 0.515 | 0.869 |  |  |  |  |  |  |  |  | 0.553 |  |  |  |  |  | 0.349 |  |  |  |  |
| **13** | *Scomberomorus cavalla* |  | 0.262 | 0.174 | 0.593 | 0.533 |  |  |  | 0.353 | 0.552 | 0.854 |  | 0.876 | 0.496 |  |  |  |  |  |  |  |  |
| **14** | Medium pelagics |  | 0.128 | 0.197 | 0.948 | 0.649 |  |  |  | 0.157 | 0.773 |  |  | 0.115 |  |  |  |  |  |  |  |  |  |
| **15** | Small pelagics | 0.177 | 0.655 | 0.159 | 0.118 | 0.374 |  |  |  | 0.235 | 0.495 | 0.389 |  | 0.643 | 0.224 |  | 0.846 |  |  | 0.466 | 0.119 |  |  |
| **16** | Carnivorous reef fishes |  | 0.957 |  | 0.125 | 0.739 | 0.382 |  | 0.531 | 0.158 |  |  |  |  | 0.192 |  |  |  |  |  | 0.676 |  |  |
| **17** | Omnivorous reef fishes |  | 0.240 |  | 1.000 | 0.158 | 0.133 |  | 0.615 | 0.158 | 0.169 |  |  |  | 0.158 |  | 0.142 |  | 0.717 |  |  |  |  |
| **18** | Demersal fishes | 0.186 | 0.173 | 0.169 |  |  |  |  |  |  |  |  | 0.656 |  |  |  |  |  |  |  |  |  |  |
| **19** | Cephalopods | 0.500 | 0.323 | 1.000 | 0.487 |  |  |  | 0.257 |  |  |  |  | 0.442 | 0.115 |  | 0.762 | 0.577 | 0.495 |  |  |  |  |
| **20** | Carnivorous zoobenthos |  | 0.223 | 0.436 | 0.732 |  | 0.162 | 0.324 | 0.820 |  |  | 0.154 | 0.363 |  | 0.758 | 0.500 | 0.984 | 0.419 | 0.813 | 0.686 |  |  |  |
| **21** | Detritivorous zoobenthos | 0.383 |  | 0.278 |  |  | 0.382 | 0.220 | 0.422 | 0.359 |  | 0.132 | 0.363 |  | 0.832 | 0.500 | 0.195 |  | 0.139 | 0.815 | 0.297 |  |  |
| **22** | Zooplankton |  |  |  |  |  |  | 0.979 | 0.333 |  |  | 0.518 |  |  |  | 0.700 | 0.240 | 0.357 | 0.713 |  | 0.217 | 0.119 |  |
| **23** | Macroalgae |  | 0.221 |  |  |  |  |  | 0.395 |  |  |  |  |  | 0.000 | 0.500 | 0.156 | 0.558 |  |  | 0.217 | 0.127 |  |
| **24** | Phytoplankton |  |  |  |  |  |  |  |  |  |  |  |  |  |  | 0.100 | 0.742 |  |  |  |  | 0.255 | 1.000 |
| **25** | Detritus |  | 0.218 | 0.170 |  |  | 0.790 | 0.649 | 0.166 |  |  |  | 0.266 |  |  | 0.500 | 0.156 | 0.320 | 0.575 |  | 0.216 | 0.499 |  |

Source of diet compounds: Dolphins [39,40,41]; Sharks [42-45]; Dogfish [46]; Large pelagics [47,43]; *Thunnus atlanticus* [48]; Grouper [49]; *Lutjanus* spp [50]; *Coryphaena hippurus* [43]; *Euthynnus alletteratus* [51]; *Scomberomorus brasiliensis, Seriola fasciata, Cynoscion jamaicensis, Scomberomorus cavala,* Medium pelagics, Small pelagics, Carnivorous reef fishes, Omnivorus reef fishes and Demersal fishes online database (fishbase.org); Cephalopods [52-54]; Carnivorous zoobenthos [55-57]; Detritivorous zoobenthos [57,58].
