# Supplementary material for: More than Anecdotes: Fishers’ Ecological Knowledge Can Fill Gaps for Ecosystem Modeling
Source: PLoS One. 2016 May 19;11(5):e0155655. doi: 10.1371/journal.pone.0155655 (PMC4873290; doi:10.1371/journal.pone.0155655)
Supplement: S3 Table — Diet matrix for the 25 compartments using Ecopath with Ecosim. The predators are set in columns and the prey is set in lines. (DOCX) [file pone.0155655.s003.docx]

**Supporting information**

**S3 Table. Fishers’ knowledge model diet matrix.** Diet matrix for the 25 compartments using Ecopath with Ecosim. The predators are set in columns and the prey is set in lines.

|  | **Prey \ predator** | **1** | **2** | **3** | **4** | **5** | **6** | **7** | **8** | **9** | **10** | **11** | **12** | **13** | **14** | **15** | **16** | **17** | **18** | **19** | **20** | **21** | **22** |
| --- | --- | --- | --- | --- | --- | --- | --- | --- | --- | --- | --- | --- | --- | --- | --- | --- | --- | --- | --- | --- | --- | --- | --- |
| **1** | Dolphin |  |  |  |  |  |  |  |  |  |  |  |  |  |  |  |  |  |  |  |  |  |  |
| **2** | Sharks |  |  |  |  |  |  |  |  |  |  |  |  |  |  |  |  |  |  |  |  |  |  |
| **3** | Dogfish |  | **0.433** |  | 0.653 |  |  |  |  |  |  |  |  |  |  |  |  |  |  |  |  |  |  |
| **4** | Large pelagics |  |  |  |  |  |  |  |  |  |  |  |  |  |  |  |  |  |  |  |  |  |  |
| **5** | *Thunnus atlanticus* |  | **0.834** |  | 0.197 |  |  |  |  |  |  |  |  |  | 0.227 |  |  |  |  |  |  |  |  |
| **6** | Groupers |  | **0.834** |  |  |  |  |  |  |  |  |  |  |  |  |  |  |  |  |  |  |  |  |
| **7** | *Scomberomorus brasiliensis* | **0.200** |  |  | 0.363 |  |  |  |  |  |  |  |  |  | 0.795 |  |  |  |  |  |  |  |  |
| **8** | *Lutjanus* spp |  | **0.948** |  | 0.653 |  |  |  |  |  |  |  |  |  |  |  | 0.136 |  |  |  |  |  |  |
| **9** | *Seriola fasciata* |  | **0.834** |  | 0.653 |  |  |  |  |  |  |  |  |  | 0.175 |  |  |  |  |  |  |  |  |
| **10** | *Coryphaena hippurus* |  | **0.834** |  | 0.122 |  |  |  |  |  |  |  |  |  | 0.284 |  |  |  |  |  |  |  |  |
| **11** | *Euthynnus alletteratus* | **0.800** | **0.533** |  | 0.916 |  |  |  |  |  |  |  |  |  | 0.455 |  |  |  |  |  |  |  |  |
| **12** | *Cynoscion jamaicensis* |  | **0.834** |  |  |  |  |  |  |  |  |  |  |  |  |  |  |  |  |  |  |  |  |
| **13** | *Scomberomorus cavalla* | **0.250** | **0.574** |  | 0.617 |  |  |  |  |  |  |  |  |  |  |  |  |  |  |  |  |  |  |
| **14** | Medium pelagics | **0.100** | **0.999** | **0.150** | 0.350 |  | **0.111** | **0.200** |  | **0.430** |  |  |  | **0.470** | 0.568 |  |  |  |  |  |  |  |  |
| **15** | Small pelagics | **0.200** | **0.733** | **0.250** | 0.122 | **0.171** | **0.150** | **0.320** | **0.142** | **0.227** | **0.667** | **0.733** | **0.200** | **0.667** | 0.227 |  | 0.678 |  |  | 0.855 | 0.118 |  |  |
| **16** | Carnivourous reef fish |  | **0.163** | **0.830** | 0.122 | **0.260** | **0.940** | **0.125** | **0.150** | **0.150** | **0.111** | **0.670** |  |  |  |  | 0.678 |  |  |  |  |  |  |
| **17** | Omnivorous reef fish |  |  |  |  | **0.200** | **0.900** | **0.125** |  |  |  |  |  |  |  |  |  |  | 0.280 |  |  |  |  |
| **18** | Demersal fish |  |  |  |  |  |  |  |  |  |  |  | **0.300** |  |  |  |  |  |  |  |  |  |  |
| **19** | Cephalopods | **0.150** | **0.322** | **0.167** | 0.122 | **0.176** |  | **0.143** | **0.125** |  | **0.111** | **0.133** |  | **0.830** | 0.568 |  | 0.678 | 0.351 | 0.600 |  |  |  |  |
| **20** | Carnivorous zoobenthos |  |  |  |  |  |  |  | **0.280** |  |  |  |  |  | 0.795 |  | 0.656 | 0.350 | 0.236 | 0.727 |  |  |  |
| **21** | Detritivorous zoobenthos |  |  | **0.830** |  | **0.118** |  | **0.479** |  |  | **0.111** | **0.670** | **0.375** | **0.830** | 0.796 |  | 0.136 | 0.950 | 0.427 | 0.300 | 0.589 |  |  |
| **22** | Zooplankton |  |  |  |  |  |  | **0.479** |  |  |  |  |  |  | 0.114 | 0.563 | 0.136 | 0.160 | 0.265 | 0.145 | 0.236 | 0.225 | 0.999 |
| **23** | Macroalgae |  |  |  |  |  |  |  |  |  |  |  |  |  |  |  | 0.700 | 0.250 |  |  |  |  |  |
| **24** | Phytoplankton |  |  |  |  |  |  |  |  |  |  |  |  |  |  | 0.375 | 0.678 | 0.500 |  |  |  | 0.225 | 0.676 |
| **25** | Detritus |  | **0.230** | **0.267** |  | **0.590** |  |  |  |  |  |  | **0.125** |  | 0.227 | 0.625 | 0.136 | 0.163 | 0.250 | 0.145 | 0.354 | 0.450 | 0.315 |

Bolded values refer to information on diet composition supplied by fishers.
